# Host immune responses associated with SARS-CoV-2 Omicron infection result in protection or pathology during reinfection depending on mouse genetic background

**DOI:** 10.21203/rs.3.rs-3637405/v1

**Published:** 2023-11-29

**Authors:** Gagandeep Singh, Prajakta Warang, Juan García-Bernalt Diego, Lauren Chang, Yonina Bykov, Sarabjot Singh, Lars Pache, Sara Cuadrado-Castano, Brett Webb, Adolfo Garcia-Sastre, Michael Schotsaert

**Affiliations:** Icahn School of Medicine at Mount Sinai; Icahn School of Medicine at Mount Sinai; Icahn School of Medicine at Mount Sinai; Icahn School of Medicine at Mount Sinai; Icahn School of Medicine at Mount Sinai; RT-PCR COVID-19 Laboratory, Civil Hospital, Moga, Punjab, India; Sanford Burnham Prebys Medical Discovery Institute; Icahn School of Medicine at Mount Sinai; Department of Veterinary Sciences, University of Wyoming; Icahn School of Medicine at Mount Sinai; Icahn School of Medicine at Mount Sinai

## Abstract

Rapid emergence of antigenic distinct SARS-CoV-2 variants implies a greater risk of reinfection as viruses can escape neutralizing antibodies induced by vaccination or previous viral exposure. Disease severity during COVID-19 depends on many variables such as age-related comorbidities, host immune status and genetic variation. The host immune response during infection with SARS-CoV-2 may contribute to disease severity, which can range from asymptomatic to severe with fatal outcome. Furthermore, the extent of host immune response activation may rely on underlying genetic predisposition for disease or protection. To address these questions, we performed immune profiling studies in mice with different genetic backgrounds - transgenic K18-hACE2 and wild-type 129S1 mice – subjected to reinfection with the severe disease-causing SARS-CoV-2 B.1.351 variant, 30 days after experimental milder BA.1 infection.

BA.1 preinfection conferred protection against B.1.351-induced morbidity in K18-hACE2 mice but aggravated disease in 129S1 mice. We found that he cytokine/chemokine profile in B.1.351 re-infected 129S1mice is similar to that during severe SARS-CoV-2 infection in humans and is characterized by a much higher level of IL-10, IL-1β, IL-18 and IFN-γ, whereas in B.1.351 re-infected K18-hACE2 mice, the cytokine profile echoes the signature of naïve mice undergoing viral infection for the first time. Interestingly, the enhanced pathology observed in 129S1 mice upon reinfection cannot be attributed to a less efficient induction of adaptive immune responses to the initial BA.1 infection, as both K18-hACE2 and 129S1 mice exhibited similar B and T cell responses at 30 DPI against BA.1, with similar anti-BA.1 or B.1.351 spike-specific ELISA binding titers, levels of germinal center B-cells, and SARS-CoV-2-Spike specific tissue-resident T-cells. Long-term effects of BA.1 infection are associated with differential transcriptional changes in bronchoalveolar lavage-derived CD11c + immune cells from K18-hACE2 and 129S1, with K18-hACE2 CD11c + cells showing a strong antiviral defense gene expression profile whereas 129S1 CD11c + cells showed a more pro-inflammatory response. In conclusion, initial infection with BA.1 induces cross-reactive adaptive immune responses in both K18-hACE2 and 129S1 mice, however the different disease outcome of reinfection seems to be driven by differential responses of CD11c + cells in the alveolar space.

## Introduction

Sever acute respiratory syndrome coronavirus 2 (SARS-CoV-2), the causative agent of the coronavirus disease 2019 (COVID-19) pandemic, has evolved into several variants with differential characteristics since it originally started circulating at the end of 2019^1^. One of the major challenges in controlling the pandemic is to better understand the risk and underlying mechanisms that allow reinfection by different variants of SARS-CoV-2, which may affect vaccine efficacy and protection provided by natural immunity^2–5^.

The immune response to SARS-CoV-2 infection is complex and influenced by both viral and host factors - viral load, duration of exposure, genetics and the presence of comorbidities have shown to be the most common pivotal factors^6–9^. Reinfection can occur as earlier as 3 months after the primary infection and it is more likely to be caused by antigenic distant variants^10,11^. Still, the correlates of protection and disease outcome from SARS-CoV-2 reinfection are yet to be determined and may involve adaptive as well as innate immune mediators. Understanding the factors that influence the immune response to SARS-CoV-2 infection and reinfection is essential for developing effective therapies and preventive strategies.

Animal models are indispensable for investigating the immunological aspects of COVID-19 and to understand the differential responses elicited by the host towards distinct variants. Wild-type mouse strains are resistant to infection with ancestral SARS-CoV-2 variants due to the low affinity of the viral spike protein to mouse angiotensin-converting enzyme 2 (mACE2), the cellular receptor for SARS-CoV-2 entry. The generation of K18-hACE2 mice, that express human ACE2 (hACE2) under the control of the mouse K18 promoter, helped to overcome this limitation in the experimental setting^12^. However, the overexpression of hACE2 in tissues other than the respiratory tract, such as the brain, increases the risk of systemic viral infection with high lethality even at low doses of inoculum^13^. SARS-CoV-2 variants that circulated later during the pandemic with N501Y mutation in their S-protein receptor binding domain (RBD) are able to infect inbred mice strains such as BALB/c, C57BL6, and 129S1^14^. SARS-CoV-1 and SARS-CoV-2 replication was observed in lungs of all three inbred mouse strains with 129S1 mice showing higher susceptibility to infection and morbidity^14–17^.

**Figure 1:**
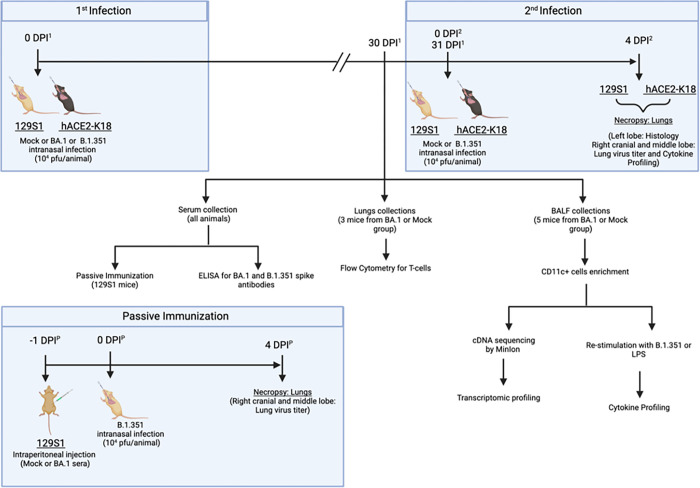
Impact of SARS-CoV-2 reinfection in mice with different genetic backgrounds, experiment design: Mice were first infected with BA.1 (104 pfus) and rested for 30 days. 31 days post –1st infection (DPI1), mice were reinfected (2nd infection) with 104 pfus of B.1.351, a dose that is lethal for naive K18-hACE2 mice and which typically causes 10–15% transient body weight loss in naive 129S1 mice. At 30 DPI^1^, BA.1 infected or mock-infected mice (N=3) were euthanized to collect whole lungs for downstream analysis of T-cell responses by flow cytometry (i), bronchoalveolar lavage fluid (BALF; N=5) was source for CD11c+ isolation (ii), CD11c+ cells were further subjected to transcriptome and cytokine/chemokine recall response analysis. Serum collected at 30 DPI^1^ was used to assess antibody responses against BA.1 and B.1.351 spike protein (i) and further used for passive immunization studies (ii): 129S1 mice were intraperitoneal injected with pooled serum from either mock infected or BA.1 infected mice, followed by infection with B.1.351 (10^4^ PFU). Mice infected with B.1.351 were necropsied at 4 DPI^1^/DPI^2^/DPI^P^ for downstream analysis involving lung virus titers, lung histopathogy and cytokine/chemokine responses.

Here, we used 129S1 and K18-hACE2 mouse strains to study the outcomes of reinfection using two different variants of SARS-CoV-2, BA.1(Omicron) and B.1.351 (Beta), both harboring the N501Y mutation in the RBD of their S-protein (Fig. 1). After initial exposure to Omicron BA.1 and after 4 weeks recovery, mice were reinfected with the B.1.351 variant at a dose that is lethal for naive K18-hACE2 mice, and which typically causes 10–15% transient body weight loss in naive 129S1 mice. We observed that prior exposure to BA.1 resulted in protection during reinfection with B.1.351 in K18-hACE2 mice. In contrast, 129S1 mice displayed increased morbidity, higher weight loss and lung damage upon reinfection. The different disease outcomes after secondary infection could not be correlated with differences in host adaptive immune responses induced after primary infection, as addressed by serology and T cell immunity. However, BA.1 infection affected the response of the alveolar CD11c + cell population during restimulation experiments, and these altered responses where further dependent on mouse genetic background. The altered responsiveness of alveolar CD11c + cells correlate with the different outcome of disease during reinfection with B.1.351 infection.

## Material and Methods

### Cells and Viruses

Vero-TMPRSS2 cells (BPS Bioscience, Cat# 78081) were cultured at 37°C in 1X DMEM (Dulbecco’s modified Eagle medium) supplemented with 10% FBS (fetal bovine serum), 100 U/ml penicillin-streptomycin and 5 μg/ml puromycin. hCoV-19/USA/NY-MSHSPSP-PV44476/2021 (GISAID: EPI_ISL_7908052) B.1.1.529 (Omicron, BA.1) and hCoV-19/USA/MD-HP01542/2021 JHU (Beta, B.1.351, a kind gift from Dr. Andy Pekosz) cultured and tittered in Vero-TMPRSS2 cells followed by next-generation sequencing to confirm the stability of the variant specific mutations and the absence of undesired mutations.

### Animal experiments

All animal experiments were approved by the Institutional Biosafety committee and by the Institutional Animal Care and Use Committee at Icahn School of Medicine at Mount Sinai (ISMMS) (PROTO202100007). All SARS-CoV-2 virus experiments were performed in a biosafety level 3 facility at the ISMMS.

Heterozygous K18-hACE2 C57BL/6J (strain 2B6.Cg-Tg(K18-ACE2)2Prlmn/J) and 129S1 (strain 129S1/SvImJ) female mice were procured from The Jackson Laboratory. The mice were housed in an animal vivarium at ISMMS with a 12-hour light-dark cycle and fed standard chow diets. Mice were transferred to the Animal biosafety level 3 facility one week prior to any virus inoculation experiments. In a first infection experiment, 3-month-old female K18-hACE2 and 129S1 mice were anaesthetized with an intraperitoneal (IP) injection of a Ketamine/Xylazine cocktail and intranasally (IN) inoculated with either 10^4^ PFU (plaque forming unit) of B.1.1.529 (BA.1) or mock media (PBS) in 50 μl. At 30 days post infection (DPI)^1^, serum samples, BALF or whole lungs were collected from mice inoculated IN with either BA.1 or mock. In a second infection experiment, the remaining mice were inoculated (IN) with 10^4^ PFU of B.1.351 or mock media under Ketamine/Xylazine cocktail anesthesia resulting into 3 groups: Mock: Mock (1st infection mock and 2nd infection mock), Mock: B.1.351 (1st infection mock and 2nd infection B.1.351), and BA.1: B.1.351 (1st infection BA.1 and 2nd infection B.1.351). At 4 DPI^2^ all mice were euthanized by pentobarbital IP injection [Figure 1].

Passive immunization: Five 3-months-old female 129S1 mice were IP injected (150μl/mouse) with sera collected either from mock or BA.1 infected 129S1 mice from the first infection. After 12hrs, mice were IN inoculated with 10^4^ PFU of B.1.351 under Ketamine/Xylazine cocktail anesthesia. At 4 DPI all mice were euthanized by pentobarbital IP injection [Figure 1].

The cranial and middle lobes of the right lung from mice were collected and homogenized in 500μl of Phosphate Buffered Saline (PBS) and used for lung viral titers and cytokine profiling, whereas left lobe of the lungs was perfused and stored with 10% neutral buffered formalin for histology.

### Humoral immune response against BA.1 or B.1.351 spike protein.

ELISA against BA.1 or B.1.351 spike was performed on serum collected at 30 DPI^1^. Nunc maxisorp 96-plates were coated with 0.1μg/well (in 50ul) of BA.1 (Sino Biologicals) or B.1.351 spike protein (Sino Biologicals) and incubated overnight at 4°C using PBS as coating buffer. The next day, plates were washed three times with PBS-Tween20 (0.1%) (PBST) and blocked for 2hrs with 2% BSA in PBST at room temperature, followed by three washes with PBST. 100μl of three-fold diluted serum staring from 1:10 were added into the wells and incubated for 2hrs at room temperature on a shaker. After three washes with PBST, 100μl of anti-mouse IgG HRP (Horse Radish Peroxidase) secondary antibodies were added in to the well and incubated for 1hr at room temperature on a shaker. Plates were washed three times with PBST, and a peroxidase reaction was catalyzed using 1-Step^™^ TMB (tetramethyl benzidine) ELISA Substrate Solutions (ThermoFisher Scientific) and incubated for 15mins at room temperature in the dark. Finally, the reaction was stopped by adding 50 μl/well of ELISA Stop Solution (ThermoFisher Scientific). The optical density (OD) readings at 450 nm were obtained using a spectrophotometer.

At 30 DPI^1^, BA.1 infected or mock-infected mice (N = 3) were euthanized to collect whole lungs for downstream analysis of T-cell responses by flow cytometry (i), bronchoalveolar lavage fluid (BALF; N = 5) was source for CD11c + isolation (ii), CD11c + cells were further subjected to transcriptome and cytokine/chemokine recall response analysis. Serum collected at 30 DPI^1^ was used to assess antibody responses against BA.1 and B.1.351 spike protein (i) and further used for passive immunization studies (ii): 129S1 mice were intraperitoneal injected with pooled serum from either mock infected or BA.1 infected mice, followed by infection with B.1.351 (10^4^ PFU). Mice infected with B.1.351 were necropsied at 4 DPI^1^/DPI^2^/DPI^P^ for downstream analysis involving lung virus titers, lung histopathogy and cytokine/chemokine responses.

### Flow cytometry

The whole lungs collected at 30 DPI^1^ were dissociated into a single cell suspension using gentleMACS^™^ Octo Dissociator (Miltenyi Biotec) and Mouse Lung Dissociation Kit, mouse (Miltenyi Biotec) as per manufacturer’s instructions. The single cell suspension was then treated with eBioscience^™^ 1X RBC Lysis Buffer (ThermoFisher Scientific) to remove erythrocytes. The treated cells were then washed twice with PBS and resuspended in eBioscience^™^ Flow Cytometry Staining Buffer (ThermoFisher Scientific) containing 1:50 Mouse BD Fc Block^™^ (BD biosciences) antibody and incubated at room temperature for 15 mins, followed by staining with anti-CD3, CD8, CD44, CD69, CD103 (BD biosciences) and SARS-CoV-2 spike tetramer (VNFNFNGL, NIH core tetramer) for 15 mins at room temperature in the dark. The cells were washed twice with eBioscience^™^ Flow Cytometry Staining Buffer (ThermoFisher Scientific) and fixed by adding 10% formaldehyde (5% final concentration in staining buffer) and incubating for 48hrs at 4°C. Fixed cells were washed twice with staining buffer and filtered through a 100μm cell strainer (Falcon) to remove clumped cells. Filtered single cell suspensions were then analyzed using the Gallios Flow Cytometer (Beckman Coulter) and FlowJo software.

### BALF CD11c + cells analysis.

CD11c + cells were enriched from all groups (n = 5/group) at 30 DPI^1^ using CD11c MicroBeads UltraPure (Miltenyi Biotec) as per manufacturer’s instructions. The pooled enriched cells were washed twice with cold RPMI-1640 medium containing 1X penicillin-streptomycin cocktail (Corning). Washed cells were plated in flat-bottom tissue culture 96-well plate (Falcon) at 200,000 cells/well. The cells were stimulated either with RPMI medium or with LPS (10ng/ml) or with 10^4^PFU of B.1.351 SARS-CoV-2 live virus for 48hrs. After 48hrs, cell supernatants were collected, UV-inactivated, and used for cytokine profiling by Luminex.

### Determination of viral titers in lungs

Cranial and middle lobes of the right lungs from both 129S1 and K18-hACE2 mice (mock: mock or mock: B.1.351 or BA.1: B.1.351 infected) were collected at 4 DPI^2^ and homogenized in sterile PBS. Homogenized lungs samples were cleared by centrifugation at 5,000g for 5 min at 4°C and subjected to plaque assays as described previously ^10^.

Briefly, twelve-well tissue culture plates were seeded with 1 × 10 Vero-TMPRSS2 cells per well. The following day, medium was removed and inoculated with 200μl of cleared lung homogenates serially diluted PBS. After 1hr, the inoculum was removed and replaced with 1% Oxoid^™^ Purified Agar (ThermoFisher Scientific) in 1xDMEM + 2% FBS overlay. Plates were incubated for 72 hrs at 37°C supplemented with 5% CO_2_, and then fixed with 5% paraformaldehyde (final concentration) in PBS overnight at 4°C. The 5% paraformaldehyde and agar overlay were carefully removed, and plates were washed once with PBST. Plates were immuno-stained using 1:1000 anti-SARS-CoV-2-N monoclonal antibody (1C7C7) on a shaker at room temperature for 1 hr followed by 1:5000 anti-mouse IgG HRP-conjugated antibody (ThermoFisher Scientific) incubated at room temperature for 1 hr. The peroxidase reaction was catalyzed using KPL TrueBlue Peroxidase Substrate (Seracare) for 15 mins at room temperature and washed with tap water. The immuno-stained plaques were counted, and titers were calculated and represented as PFU/ml.

### Cytokines and chemokines multiplex analysis

The cranial and middle lobes of the right lungs from both 129S1 and K18-hACE2 mice K18-hACE2 mice (mock: mock or mock: B.1.351 or BA.1: B.1.351 infected) were collected at 4 DPI^2^, homogenized. Homogenized lungs samples were centrifuged at 5,000×g for 5 min at 4°C and transferred into clear-U-bottom 96-well plate (Falcon). Samples were inactivated with ultraviolent (UV-C) light for 15 mins on ice. Inactivated samples were then used to profile cytokine and chemokine levels using a mouse 26-plex, bead-based Luminex assay (catalogue number EPXR260-26088-901). The assay was performed according to the manufacturer’s instructions, and all incubation steps occurred on an orbital shaker set at 300 rpm. Briefly, 50 μl of clarified lung homogenate supernatant was combined with beads in a lidded, black 96-well plate supplied as part of the kit and incubated for 30 min at room temperature, and then overnight at 4°C. The next day, the plate was allowed to equilibrate to room temperature for 30 min, washed 3 times with 150 μl per well of 1× wash buffer, and then 25 μl per well of 1× detection antibody mixture was added for 30 min at room temperature. The plate was washed 3 times, and then 50 μl per well of 1× Streptavidin–PE solution was added for 30 min at room temperature. After washing 3 times, 120 μl per well of reading buffer was added, and the plate was incubated for 5 min at room temperature. Data were acquired on a Luminex 100/200 analyzer (Millipore) with xPONENT software (version 4.3) and analyzed using GraphPad Prism (version 8.0) and R (version 4.0.5).

### Pathology

The left lobe of the lungs was perfused and fixed in 10% formaldehyde in PBS. After 4 weeks, 10% formaldehyde was replaced with 1X PBS and shipped to Wyoming State Veterinary Laboratory (Laramie, WY) for tissue processing and histology examination. The H&E-stained slides were blindly scored by a Board-Certified Veterinary Pathologist. Lung tissue sections were scored as per Table 1.

### Long read transcriptome profiling

The pooled enriched cells were also used for transcriptome profiling of CD11c + cells. The RNA from enriched cells was isolated using Trizol, and mRNA was enriched using Next Poly (A) mRNA Magnetic Isolation Module (NEB) as per manufacturer’s instructions. One nanogram of purified mRNA then used to prepare cDNA library using PCR-cDNA Sequencing Kit (Oxford Nanopore) as per manufactures’ instructions. RNA sequencing was then performed on the MinION MK1b device (Oxford Nanopore) using FLO-MIN106D Flow cells (Oxford Nanopore).

The output reads were analyzed using Minimap2, Samtools, and Stringtie packages^18–20^. The normalized final counts then were analyzed and visualized using R packages (ggsankey) and Graphpad Prism^21^.

### Statistical analysis

Unpaired t-tests analysis was used for determining the Statistical significance between experimental groups, where p-values > 0.05 are denoted as ‘ns’, 0.05 > 0.01 as ‘ * ’, 0.01 > 0.001 as ‘ ** ’.The number of technical replicates are noted in figure legends.

## Results

### Prior exposure to BA.1 affects disease severity outcomes in K18-hACE2 in 129S1 mice undergoing B.1.351 reinfection.

Reinfections with SARS-CoV-2 have been widely reported, especially since the emergence of antigenically drifted Omicron lineages able to escape neutralizing antibodies induced by previous infection or vaccination^2,4,5^. The outcome of the second infection has shown to be influenced by the antigenic match and quality of host immune responses induced by the first infection, ranging from full protection to partial protection ^2,4,5^.

**Figure 2:**
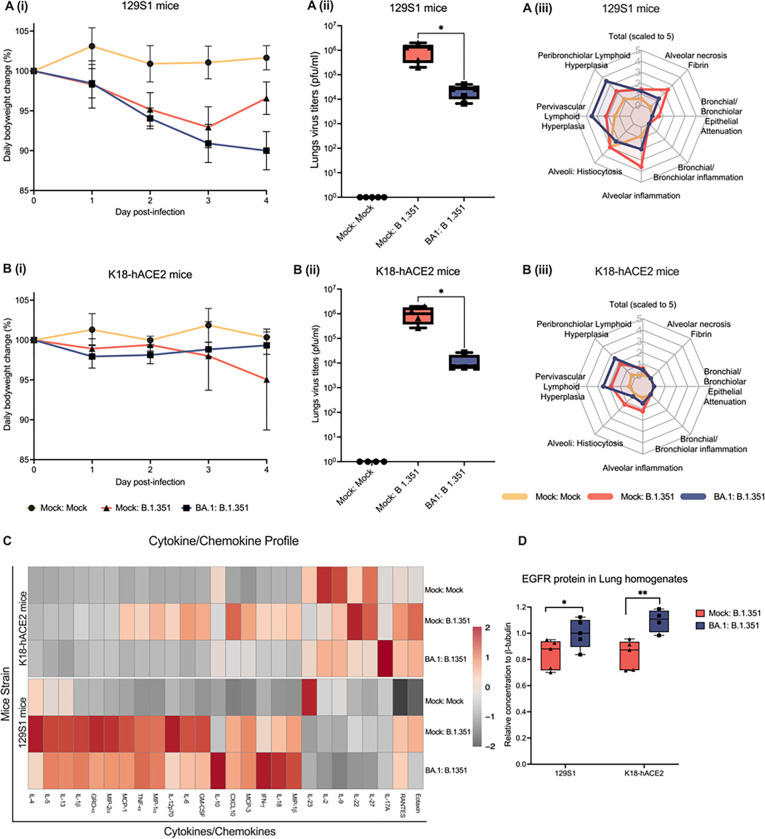
Pathology associated to B.1.351 infection in BA.1 preinfected mice. K18-hACE2 or 129S1 mice were reinfected with B.1.351 30 days after initial viral exposure to Omicron BA.1 variant. A (i): Weight change in mock or BA.1 pre-infected 129S1 mice (n=5) inoculated with either mock or 10^4^ pfu of B.1.351. A (ii): Infectious virus titers in the lungs of mock or BA.1 pre-infected 129S1 mice (n=5) inoculated with either mock or 10^4^ pfu of B.1.351. A (iii): Radar graph showing histopathological scores from mock or BA.1 pre-infected 129S1 mice (n=5) inoculated with either mock or 10^4^ pfu of B.1.351. B (i): Weight change in mock or BA.1 pre-infected K18-hACE2 mice (n=5) inoculated with either mock or 10^4^ pfu of B.1.351. B (ii): Infectious virus titer in lungs of mock or BA.1 pre-infected K18-hACE2 mice (n=4) mice inoculated with either mock or 10^4^ pfu of B.1.351. B (iii): Radar graph of the histopathological scores from mock or BA.1 pre-infected K18-hACE2 mice (n=5) inoculated with either mock or 10^4^ pfu of B.1.351. C: Heatmap graph of cytokine/chemokine profile of lung homogenates from mock or BA.1 pre-infected K18-hACE2 (n=4) or 129S1 (n=5) mice inoculated with either mock or 10^4^ pfu of B.1.351. D: EGFR protein quantification from lung homogenates from BA.1 pre-infected K18-hACE2 (n=4 or 5) or 129S1 (n=5) mice inoculated with either mock or 10^4^ pfu of B.1.351. Two-tailed t-test; p-value > 0.05 ‘ns’, 0.05 > 0.01 ‘ * ’, 0.01 > 0.001 ‘ ** ’.

Here, we tested wether mice that went through omicron BA.1 infection would be protected from severe morbidity during B.1351 reinfection. The selection of these two SARS-CoV-2 variants was based on our previous results demonstrating that BA.1 infection is mild and causes no morbidity in mice whereas B.1.351 infection can cause severe morbidity in mouse models and is lethal in the K18-hACE2 mice model ^13^. Reinfection of 129S1 and K18-hACE2 mice was carried out 30 days after first infection with 10^4^ pfu of mild morbidity-causing SARS-CoV-2 Omicron BA.1 variant [Figure 1]. In both mouse models, B.1.351 reinfected mice showed a 1.5 log_10_ reduction in lungs virus titers compared to the first time-infected groups [Figure 2A (ii) and 2B (ii)]. However, we observed contrasting results in daily body weight data, with B.1.351 reinfected K18-hACE2 mice displaying protection while 129S1 mice showed signs of severe morbidity as measured by body weight loss after B.1.351 infection [Figure A (i) and B (i)].

Histopathological examination of lungs from reinfected mice confirmed higher pathological score for 129S1 than K18-hACE2 mice [Figure 2A (iii) and 2B (iii)]. In both mouse models, B.1.351 infection promoted inflammation and histiocytosis at the alveolar compartment, which was less severe in B.1.351 reinfected mice. B.1.351 infection also caused necrosis in 129S1. Alveolar inflammation and histiocytosis were present in mock infected 129S1 suggesting a distinct basal inflammatory response intrinsic / inherent to the genetic background of these mice. In contrast to the above results, we observed that B.1.351 reinfection in BA.1 infected mice results in enhanced peribronchial and perivascular lymphoid hyperplasia [Figure A (iii) and B (iii)]. On closer examination,129S1 mice shown higher presence of macrophages with eosinophilic cytoplasm; this was true for all the experimental 129S1 groups irrespectively of any prior infection, unveiling itself as a distinct hallmark of 129S1 mice inflammatory response [supplementary Fig. 1A]. Altoghether, 129S1 mice and K18-hACE2 mice seem to have different baseline levels of inflammation, as well as composition of the alveolar macrophage compartment.

In addition to the distinct pathology, strain-specific differences were also identifIied in the cytokine profile displayed by K18-hACE2 and 129S1 mice [Figure 2C]: B.1.351-only infection of K18-hACE2 mice propmted a Th1-like immune responses characterized by high levels of CXCL10, IFN-γ, IL-12p70, GM-CSF, RANTES, TNF-α, MIP-1 α, MCP-3, MCP-1, IL-18 and IL-6. Levels of these cytokine/chemokines were found to be similar to those exerted by mock-infected animals in the BA.1 pre-infected K18-hACE2 mice [Supplementary Fig. 1A]. interestingly, while detection of IL-17A was comparable to mock in B.1.351-only infection, we found higher IL-17A levels in BA.1 pre-infected B.1.351 infected K18-hACE2 mice. In contrast to the responses observed in K18-hACE2 mice, B.1.351-only infection elicited both Th1 and Th2 immune response in 129S1[Figure 2C, supplementary Fig. 1B], Upon reinfection, 129S1 mice displayed a raise in the expression of IL-10, IL-18 and IFN-γ.

The overexpression of Epidermal Growth Factor Receptor (EGFR) and associated signaling pathways has also been reported to correlate with severe disease and enhanced fibrosis during SARS-CoV infection^22^. To determine whether enhanced EGFR expression levels were associated with the pathological cases observed in our mice models, we analyzed the EGFR protein content in lung homogenates from B.1.351 re-infected and first-time infected mice. In both K18-hACE2 and 129S1 mice, reinfection with B.1.351 led to higher levels of EGFR when compared to mice that were infected in the absence of prior virus exposure [Figure 2D].

### 129S1 and K18-hACE2 mice exhibit similar adaptive immune responses against BA.1 infection.

**Figure 3:**
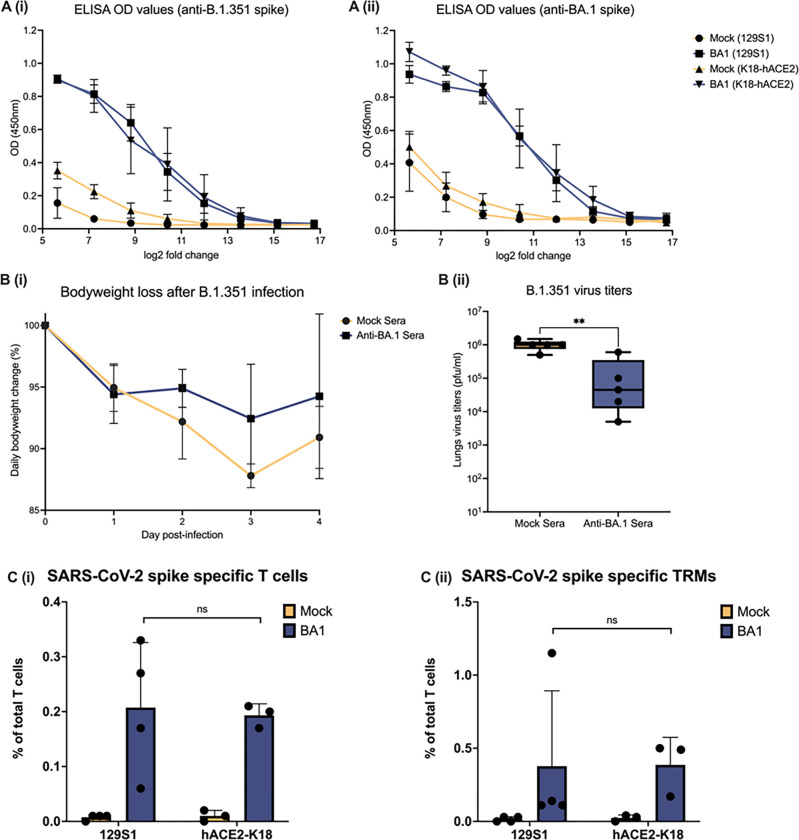
Adaptive immune responses upon BA.1 infection at 30 DPI. A: Line graph showing ELISA results in transformed log2 fold dilutions (x-axis) of 30 DPI serum from K18-hACE2 (n=4) and 129S1 (n=5) versus OD values at 450nm (y-axis) against B.1.351 spike (i) or BA.1 spike (ii) protein. B (i): Weight change in 129S1 mice inoculated with 10^4^ PFU of B.1.351 after passive immunization with either mock (n=5) or ant-BA.1 serum (n=5). B (ii): Infectious virus titers in lungs from passive immunized 129S1 mice B.1.351 inoculation. C (i): Spike-specific T-cells as percentage of total T cells in murine lungs at 30 DPI with BA.1 or mock. C (ii): Spike-specific tissue resident memory T-cells as percentage of total T cells in murine lungs at 30 DPI with BA.1 or mock. Two tailed t-test was used to determine statistical significance of the results, where p-values > 0.05 are denoted as ‘ns’, 0.05 > 0.01 as ‘ * ’, 0.01 > 0.001 as ‘ ** ’.

Next, we investigated wether the genetic background could affect the adaptive immune responses against infection in our experimental setup. After BA.1 infection, humoral and cellular adaptive responses were assessed by ELISA and flow cytometry after, respectively. ELISA results revealed that BA.1 infection elicited comparable antibody responses against BA.1- and B.1.351- spike protein in both 129S1 and K18-hACE2 genetic backgrounds [Figure 3A (i) and (ii); Supplementary Fig. 2]. We then postulated that differences in morbidity and pathology might be due to the induction of cross-reactive but non-neutralizing anti-SARS-CoV-2 antibodies in 129S1 mice and, to a lesser extend, in K18-hACE2 mice. Therefore, we repeated the B.1.351 infection experiment in naïve 129S1 or K18-hACE2 mice after passive transfer of serum from BA.1 or mock-infected animals [Figure 1]. Interestingly, passive transfer of anti-BA.1 immune serum into naïve 129S1 mice conferred protection against severe disease [Figure 3B (i)] after B.1.351 infection, presenting similar ~ 1.5 log10 reduction in lungs viral titers as in BA.1 pre-infected mice [Figure.3B (ii)]

Flow cytometry analysis of the T cell compartment revealed that mice from both genetic backgrounds had similar numbers of SARS-CoV-2 spike-specific CD8 + T-cells and tissue resident memory CD8 + T-cells (TRMs, CD103 + CD69 + tetramer+) in total digested lungs [Figure 3C (i) and (ii)].

### CD11c + cells from the alveolar compartment react differently during ex vivo restimulation depending on mouse genetic background and prior exposure to BA.1 virus.

Unresolved inflammation at the alveolar space seems to be the major driver of morbidity and differential pathology observed between 129S1 and K18-hACE2 mice. Our lung histological analysis showed that 129S1 mice harbored macrophages with abundant eosinophilic cytoplasm in the alveolar spaces of the lungs, those been distinct and absent in K18-hACE2 mice [supplementary Fig. 1]. Moreover, exposure to respiratory viruses can result in remodeling of the lung myeloid compartment and priming of the alveolar macrophages to respond differently to new innate stimuli^23–25^.

**Figure 4:**
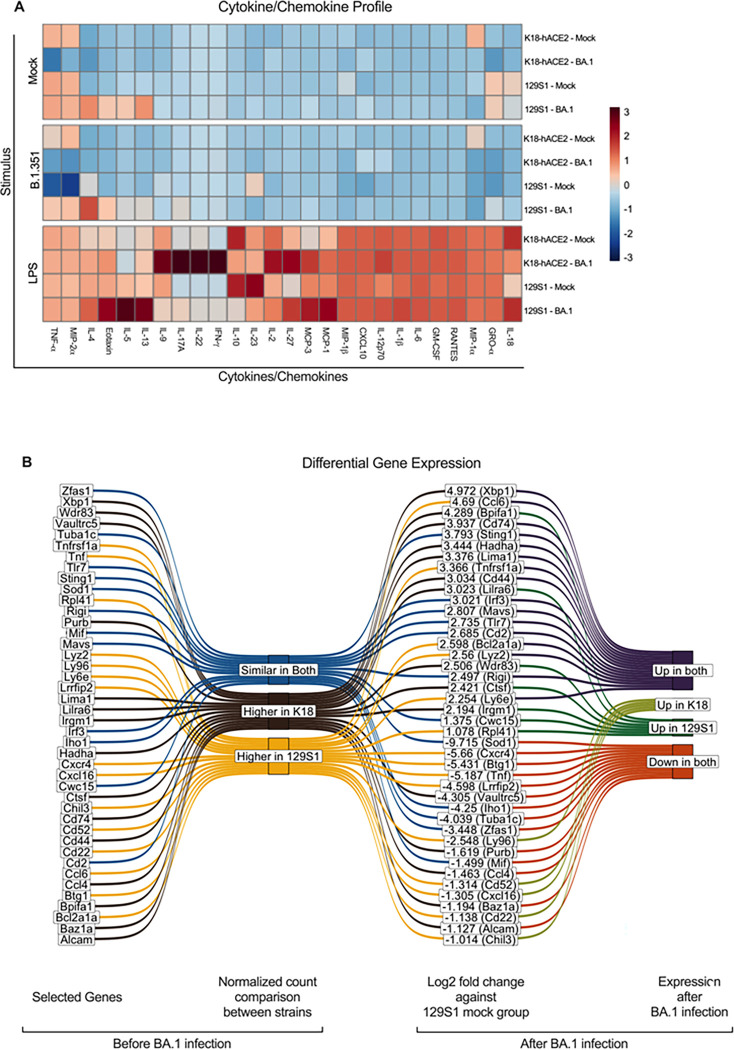
Immunological profile of murine lungs 30 days post-BA.1 infection. Mock inoculated K18-hACE2 (n=8) or 129S1 (n=9) mice were euthanized after 30 days post-infection, Lungs (n=4 or 3) or BALF (n=5) were collected. CD11c+ cells were enriched from the BALF and used for trained immunity experiment. A) Heatmap graph of the cytokine/chemokine profile of CD11c+ BALF cells either stimulated with mock media (1X RPMI, upper), Beta (10^4^ PFU of B.1.351, middle), or LPS (lower) after 48 hrs. B) Results from the long-read-RNAseq analysis from CD11c+ BALF cells isolated from mice at 30 days post-infection. Sankey diagram selected gene with their relative gene expression in mock groups and their relative change after BA.1 infection in each strain.

Therefore, we next focused on investigating whether alveolar macrophages are pivotal on the distinct immune responses observed in 129S1 and K18-hACE2 mice. To this end, CD11c + cells were isolated from the BALF of infected mice 30 days after exposure to the BA.1 variant [Figure 1]. Pooled CD11c^+^ cells were next stimulated ex vivo with either10^4^ PFU of live SARS-CoV-2 B.1.351 variant, 10 ng/ml of LPS or mock stimulated. Downstream analysis of cytokine/chemokine responses from CD11c^+^ cells revealed differences associated to genetic background both at baseline as well as after BA.1 exposure [Figure 4A].

The levels of TNF-α were high in mock groups of both models, yet the concentration in 129S1 was almost 2 times higher than that of K18-hACE2 mice. These TNF-α levels in both mouse models dropped when stimulated with the B.1.351 variant, where the 129S1 group again showed ~ 10 times reduction and K18-hACE2 only ~ 2 times reduction. When comparing mock-infected groups to BA.1 infected groups, TNF-α levels were also observed to be lower, ~ 5 times reduction in K18-hACE2 and ~ 2 times reduction in 129S1 [Figure 4A, supplementary Fig. 3A]. The levels of MIP-2 α were also elevated and were similar in the mock groups of both models (~ 1500 pg/ml), however only in 129S1 these levels dropped ~ 5-fold when stimulated with B.1.351 variant [supplementary data]. Conversely, in BA.1 infected groups, the level of MIP-2 α was lower in the K18-hACE2 group compared to the K18-hACE2 mock infected group as well as the 129S1 BA.1 infected group [Figure 4A, supplementary Fig. 3A]. Surprisingly we observed that IFN-γ, IL-17A, IL-22 was only induced by LPS stimulation in BA.1 infected K18-hACE2. IL-13 and Il-18 were only high in 129S1 mock group and were upregulated in the BA.1 infected group. However, these levels were reduced with B.1.351 stimulation, whereas LPS boosted production in all groups. BA.1 infected 129S1 CD11c + BALF cells also showed higher levels of Eotaxin when compared to other groups [Figure 4A, supplementary data].

Transcriptome profiling of CD11c + cells from BALFs also revealed differential gene expression between both mouse strains. Gene enrichment analysis assigned cells from both BA.1 infected mouse strains as M1 macrophages, 129S1 cells were classified more like RAW 264 and osteoblasts cell genotype whereas cells from K18-hACE2 showed more similarity to CD8 + T-cells and B220 + B-cell genotypes [supplementary Fig. 3B]. GO (Gene ontology analysis) biological process also showed contrasting results, where cells from K18-hACE2 showed positively regulation of the immune response, and 129S1 cells showed negative regulation of the immune response. Cells from both strains expressed a similar level of *Irf3* (interferon regulatory factor 3), *Sting1* (stimulator of interferon genes 1), *Mavs* (mitochondrial antiviral signaling protein), *Cwc15* (CWC15 spliceosome associated protein homolog), *RigI* (retinoic acid inducible gene I), *Tlr7* (toll-like receptor 7), *Tlr8* (toll-like receptor 8), *Cd2* (CD2 molecule), *Mif* (macrophage migration inhibitory factor), *Iho1* (inositol hexakisphosphate and diphosphoinositol-pentakisphosphate kinase 1), *Sod1* (superoxide dismutase 1), *Tuba1c* (tubulin α 1c), *Zfas1* (zinc finger antisense 1) genes in mock groups, out of which *Irf3, Sting1, Mavs, Rigi, Tlr7, Tlr8, Cd2* were upregulated in BA.1 infected animals, whereas *Mif, Iho1, Sod1, Tuba1c, Zfas1* were downregulated. The *cwc15* gene was upregulated in BA.1 infected 129S1 but was downregulated in BA.1 infected K18-hACE2. Compared to K18-hACE2, CD11c + BALF cells from 129S1 mice expressed higher levels of *Lyz2* (Lysozyme 2), *Ccl6* (C-C motif chemokine ligand 6), *Ly6e* (lymphocyte antigen 6 complex locus E), *Cxcr4* (C-X-C motif chemokine receptor 4), *Tnf* (tumor necrosis factor), *Lrrfip2* (leucine-rich repeat flightless-interacting protein 2), *Btg1* (B-cell translocation gene 1), *Ly9a* (lymphocyte antigen 9a), *Cd52* (CD52 antigen), *Bcl2a1a* (BCL2 related protein A1a), *Cxcr4* (C-X-C motif chemokine receptor 4), *Cd22* (CD22 antigen) and *Tnfrsf1a* (tumor necrosis factor receptor superfamily member 1A), and *Cxcl16* (C-X-C motif chemokine ligand 16). Among these genes, *Lyz2, Ccl6, Ly6e, Bcl2a1a* and Tnfrsf1a were upregulated and *Cxcr4, Tnf, Lrrfip2* and *Btg1* were downregulated by BA.1 infection in both strains. In addition, *Cxcl16, Ly96, Cd52* and *Cd22* were downregulated in 129S1 but upregulated in BA.1 infected K18-hACE2 cells. Compared to 129S1, K18-hACE2 expressed higher levels of *Alcam* (activated leukocyte cell adhesion molecule), *Ccl4* (chemokine ligand 4), *Wdr83* (WD repeat domain 83), *Bpifa1* (bactericidal/permeability-increasing fold-containing family A member 1), *Cd44* (CD44 molecule), *Lima1* (LIM domain and actin binding 1), *Xbp1* (X-box binding protein 1), and *Cd74* (CD74 molecule). Among these genes, *Alcam* and *Ccl4* were downregulated in both BA.1 infected groups of cells. *Wdr83* and *Bpifa1* were down-regulated in K18-hACE2 BA.1 infected group and up-regulated in 129S1 BA.1 infected group. *Cd44, Lima1, Xbp1*, and *Cd74* were upregulated in both BA.1 infected groups of cells.

In conclusion, different transcriptomic responses are observed in CD11c + alveolar cells upon restimulation depending on mouse genetic background and prior virus exposure. Our work describes a method to explore genetic drivers of protection or disease during SARS-CoV-2 infection in the context of reinfection.

## Discussion

Rapid evolution of SARS-CoV-2 into antigenically different variants poses a greater risk of reinfection with new variants. The severity of the reinfection depends on several factors such as antigenic distance of the variants, age, sex, comorbidities, and risk of high exposure^1^. Here, we used K18-hACE2 and 129S1 mice, two mouse strains that we have previously shown to be suitable for SARS-CoV-2 studies, to evaluate the protection and immune response after reinfection with a severe variant^14^. These strains have several differences between quantitative trait loci (QTLs) which can result in different phenotypes under certain conditions^30–33^. 129S1 mice are known to display more airway responsiveness, more airway cellular infiltration and high IL-4 levels after inhaled ovalbumin compared to the C57BL/6J strain which is of a similar genetic background to the K18-hACE2 mice^34^. 129S1 mice also have been shown to express higher levels of guanylate binding proteins (GBPs) compared to C57BL/6J when stimulated with different antigens^35^. In humans and mice, GBPs promote inflammation and restrict the replication of intracellular pathogens^35–38^. One of the main differences between the two strains is that K18-hACE2 can be infected with any SARS-CoV2 variant, 129S1 can only be infected with variants having N501Y mutation in spike (S) protein, which is also required for infection in wild type mice. Keeping this in mind, we use BA.1 and B.1.351 variants, both of which harbor the N501Y mutation in the S-protein^14^. Both variants are also antigenically different with only 93.30% similarity in the receptor binding domain (RBD) of S-protein^39^. We hypothesized that prior BA.1 infection would be mild and induce cross-reactive immune responses that are protective during reinfection with a more virulent and antigenically drifted SARS-CoV-2 virus (B.1.351). Although B.1.351 reinfection after BA.1 exposure may not occur in real life, as the B.1.351 circulated in the human population before the appearance of the SARS-CoV-2 omicron lineage that includes BA.1, we chose to continue with this experimental setup to provide proof of principle that a mild infection (BA.1) followed by a severe infection (B.1.351) may have different outcomes based on genetic background^13^.

Prior BA.1 infection was able to reduce the viral load in both strains, however, the morbidity, cytokine profile, and histopathology scoring were different based on genetic background. In K18-hACE2, prior BA.1 infection provided protection against morbidity and a cytokine profile that reflects milder disease severity. Whereas in 129S1, prior BA.1 infection exacerbated the outcome, with a cytokine profile that is somewhat similar to what is described for severe COVID-19 infection in humans with enhanced levels of IL-10, IL-18 and IFN-γ suggesting dysregulation of cytokines after BA.1 infection^40–45^. Lung pathology was assessed at 4 DPI. The difference in lung pathology at a later time point can be due to different degrees of repair after lung tissue injury upon infection, which can vary due to genetic background differences as well. Whereas controlled series of immune events can reduce lung injury and pathology, dysfunctional immune events can lead to fibrosis and scarring resulting in reduced lung function and hence quality of life^22^. The persistent damage from infectious viruses such as SARS-CoV and hepatitis C virus can lead to dysfunctional EGFR levels thus leading to more fibrosis and Type II alveolar cell hyperplasia^22^. B.1.351 infection promoted alveolar fibrosis in 129S1 only, which was reduced in mice with prior BA.1 infection. However, EGFR levels in both mice strains were similar. Interestingly, the B.1.351 reinfected mice from both genetic backgrounds showed higher EFGR levels compared to first-time infected, which was not reflected in the pathological analysis for fibrosis. As reported in humans for some viruses like respiratory syncytial virus, and a problem in the vaccination field, cross-reactive antibodies with low neutralization capacity can increase the risk of severe disease and even death^46^. This seems unlikely in our model since we performed a passive immunization experiment where we obtained similar antibody titers in both strains and passive immunization protects the 129S1, rather than exacerbated the disease. T-cells can contribute to immunopathology by causing cellular damage while targeting infected cells, but this is also typically associated with viral load reduction^47^. Alternatively, reduced T -cell numbers can decrease the viral clearance^48^. Nevertheless, the percentage of T-cells were similar in both models, and viral reduction was seen in BA.1-exposed mice in both strains. This suggests that the distinct outcomes upon reinfection are not because of differences in B- or T-cell responses.

Similarly to the adaptive immune response, the innate immune response can become more responsive after the first infection which involves epigenetic and metabolic alterations after the first infection/insult, referred as ‘trained immunity’^49^. With well-guided trained immunity, responses to a second, antigenically similar or different, infection can be protective, whereas with misguided training, responses can be deleterious^49^. To determine whether the difference is due to discrepancies in trained immunity of the innate immune cells, we enriched CD11c + cells from the BALF of mock or BA.1 infected mice, which contains 80–90% of the alveolar macrophages^50^. During histological comparison of lungs at baseline (no infection), mouse strain differences could already be observed. 129S1 mice had macrophages with abundant eosinophilic cytoplasm and a higher baseline inflammation compared to K18-hACE2 mice. Abundant eosinophilic cytoplasm of macrophages can be due to eosinophil efferocytosis, which may also have skewed the immune response to Th2 type^51–54^. We and others have also observed that prior infection can result in lung remodeling, whereby embryonic alveolar macrophages in the lung are (partially) replaced by infiltrating bone marrow-derived monocytes that upregulate alveolar macrophage markers (CD11c, SiglecF) and become lung resident^24^. As such, these recruited myeloid cells can also contribute to altered responses to stimuli during reinfection or *ex vivo* restimulation. Finally, CD11c in the mouse lung is also expressed by dendritic cells and to some extent by other (lymphoid) cell types. Therefore, isolation of CD11c + cells from the BALF results in a pool of cells that is rather heterogenous, especially when mice have been exposed before to BA.1 virus. The cytokine/chemokine profile of CD11c + cells from BA.1-exposed mice revealed that BA.1 infection is modulating the cells differently in both strains, where K18-hACE2 mice cells exhibit a more protective Type 1-like response, while 129S1 cells showed the inflammatory Type 2 response. This reflects what is already known from host-pathogen studies using these mouse strains. The CD11c + cells from the BA.1 pre-infected 129S1 showed persistent production of IL-13 even with mock stimulation, which peaked with LPS stimulation. IL-13 levels were higher in *in-vivo* B.1.351 infected 129S1 as well.

Persistent level of IL-13 can cause eosinophilic inflammatory responses resulting in lymphoid hyperplasia, airway fibrosis, and IL-5 and eotaxin production^55–57^. Our histology data showed a similar pattern, having more lymphoid hyperplasia in 129S1 compared to the K18-hACE2. The RNAseq also confirmed the up-regulation of *Mmp12* (matrix metalloproteinases 12, MMP12) and *Retnla* (resistin-like α, RELMα) gene which can be up-regulated by IL-4 and IL-13. After infection, the macrophage-produced MMP12 protein can suppress the expression of the MMP2, MMP9 and MMP13 collagenolytic proteins which results in diminishing matrix degradation, hence amplified fibrotic responses^58^. The transcriptomic analysis further supported the hypothesis that myeloid compartments are affected by prior BA.1 infection and are different in both mouse strains. RELMα secreted from activated macrophages plays a critical role in modulating type 2 cytokine production^57–59^. BA.1 infected 129S1 mice their CD11c + cells also have higher level of *Arg1* (Arginase 1) gene copies. Arginase 1, which is a marker of M2 (immunosuppressive) macrophages, can also be upregulated by IL-4 and IL-13, and is critical for cell proliferation and collagen synthesis eventually causing hyperplasia and fibrosis^60,61^.

BA.1 preinfected 129S1 also showed higher levels of IL-10, which is typically classified as an immunosuppressive and anti-inflammatory cytokine. However, there are several studies where IL-10 enhances the immune cell proliferation and activation resulting in production of pro-inflammatory cytokines^62–65^. Overexpression of IL-10 alongside with persistent IL-13 expression augments airway fibrosis, mucus metaplasia, and tissue inflammation^66^.

IFN-γ levels upon B.1.351 infection were high in BA.1 pre-infected 129S1, however this was opposite in the CD11c + cell restimulation experiment, where IFN-γ was only produced in LPS stimulated BA.1 pre-infected K18-hACE2 CD11c + cells suggesting BA.1 modulated IFN-γ gene expression in K18-hACE2 differently. Even though IFN-γ is a proinflammatory cytokine, it can inhibit IL-13’s inflammatory response and eotaxin, and protect against immunopathology which can be helpful during reinfection^67,68^. The higher IFN-γ level in lung homogenates after B.1.351 infection of BA.1 pre-infected 129S1 can be explained by persistent IL-18 expression by CD11c + cells after BA.1 infection in conjunction with IL-10 production during B.1.351 infection, as both these cytokines induce IFN-γ production in T-cells and Natural killer (NK) cells^69^. B.1.351 infection in 129S1 also induced significant amounts of another pro-inflammatory cytokine, TNF-α. CD11c + cells from both strains produced some level of TNF-α even without any stimulation, with 129S1 mice producing more. This is also evident in the transcriptome profile, where 129S1 expressed more *Tnf* gene copies than hACE2-K18. The higher production of TNF-α is further evidenced by higher counts of *Tnfaip2* gene (TNF-α induced protein 2) compared to the K18-hACE2. However, the level of TNF-α dropped in B.1.351 stimulated as well as BA.1 exposed CD11c + cells. This reduction was also observed in the transcriptome profile of 129S1, where *Tnfaip2* gene expression was downregulated. Several intracellular pathogens can downregulate TNF-α production by macrophages, which can lead to an immunosuppressive condition that favors the replication of intracellular pathogens^70,71^ .

CD11c + cells from BA.1 infected K18-hACE2 also produce high levels of IL-17 and IL-22 during LPS restimulation, with little or no change during *in vivo* B.1.351 infection. Both IL-17 and IL-22 play a significant roles in maintaining mucosal immunity via neutrophil recruitment to the infection site, up-production of antimicrobial proteins, and repair of the mucosal epithelial cell layer^72^.

The differences and similarities between the CD11c + cells from BA.1 exposed mice from different genetic backgrounds were also observed in the gene expression data. CD11c + cells were classified as M-1 macrophages after gene enrichment classification. However, while genetic profiles from 129S1 CD11c + cells are like those of other innate immune cells such as osteoclasts, microglia, and RAW cells, K18-hACE2 cells transcriptomes were more indicative of adaptive immune response components such as follicular B-cells, b220 + B-cells and CD8 + T-cells. Of note, no gene counts for surface markers CD3e, CD4, CD8a, CD16 were found in any sample and the majority of B and T cells would have been removed by positive selection of CD11c + cells, unless they would express (low levels of) CD11c.

Our findings may support the previous finding that alveolar macrophages can be considered as a source of the “primary and altered cytokine” storm induced by SARS-CoV-2 infection^48,73^. Our findings also show that different genetic backgrounds affect the immune phenotype of virus-exposed innate immune cells, which may either be beneficial or deleterious during secondary infection, and provides a starting point to explore underlying genetic differences that can affect the host response to secondary SARS-CoV-2 infection in the mouse model.

## Figures and Tables

**Table 1:** Scoring matrix for the histology examination.

Score	Area affected	Epithelial degeneration/necrosis	Inflammation
0	none	None	None
1	5–10%	Minimal; scattered cell necrosis/vacuolation affecting 5 to 10% of tissue section	Minimal; scattered inflammatory cells affecting 5–10% of tissue section
2	10–25%	Mild; scattered cell necrosis/vacuolation	Multifocal, few inflammatory cells
3	25–50%	Moderate; multifocal vacuolation or sloughed/necrotic cells	Thin layer of cells (< 5 cell layer thick)
4	50–75%	Marked; multifocal/segmental necrosis, epithelial loss/effacement	Thick layer of cells (> 5 cell layer thick)
5	>75%	Severe; coalescing areas of necrosis, parenchymal effacement	Confluent areas of inflammation

## Data Availability

Sequencing data of the will be deposited to public repository. No code was generated for this work. Unique biological materials generated for the work presented in this manuscript are available upon request for research purposes and will be shared according to standard material transfer agreements between research institutes. Requests for a material transfer agreement (MTA) can be addressed to Michael Schotsaert at Michael.Schotsaert@mssm.edu. Source data will be provided with this paper.
